# Clonal Hematopoiesis and CAR T Cell Therapy: From Biological Crosstalk to Clinical Implications

**DOI:** 10.1111/ijlh.70181

**Published:** 2026-06-22

**Authors:** Wei Du, Shunsuke Koga, Guang Yang, Saar Gill, Adam Bagg

**Affiliations:** ^1^ Department of Pathology and Laboratory Medicine Hospital of the University of Pennsylvania Philadelphia Pennsylvania USA; ^2^ Division of Hematology and Oncology, Abramson Cancer Center University of Pennsylvania Perelman School of Medicine Philadelphia Pennsylvania USA

## Abstract

Clonal hematopoiesis (CH) is increasingly recognized as a significant biological phenomenon in aging and cancer, marked by the expansion of hematopoietic stem and progenitor cells harboring somatic mutations in genes associated with myeloid neoplasms. While CH is strongly linked to a spectrum of inflammatory diseases and hematologic malignancies, its role in shaping responses to cancer immunotherapy—especially chimeric antigen receptor (CAR) T cell therapy—has only recently begun to emerge. This review explores the interplay between CH and CAR T cell therapy, highlighting CH prevalence in treated populations, post‐therapy clonal dynamics, inflammatory toxicities, and risk of therapy‐related myeloid neoplasms. We also discuss the differential effects of specific CH mutations on hematopoiesis, immune cell function, and CAR T cell persistence. Understanding the functional implications of CH in the context of CAR T cell therapy holds the potential to refine patient selection, tailor toxicity management, and develop personalized immunotherapeutic approaches.

## 1 Introduction

Chimeric antigen receptor (CAR) T cell therapy has transformed outcomes for patients with relapsed or refractory hematologic malignancies [1]. By redirecting autologous T cells toward tumor associated antigens, it achieves durable remissions in patients with previously intractable diseases such as diffuse large B‐cell lymphoma (DLBCL) [2], mantle cell lymphoma [3], follicular lymphoma [4], B‐cell acute lymphoblastic leukemia [5], and multiple myeloma [6]. Despite these successes, the CAR T cell therapy carries notable risks: cytokine release syndrome (CRS), immune effector cell‐associated neurotoxicity syndrome (ICANS), prolonged cytopenias, and late complications such as therapy‐related myeloid neoplasms (t‐MNs), which most likely reflect prior cytotoxic chemoradiation therapy and/or pre‐existing clonal hematopoiesis (CH) rather than the CAR T cell therapy itself, and very rare secondary T cell lymphomas [7, 8, 9, 10].

**FIGURE 1:**
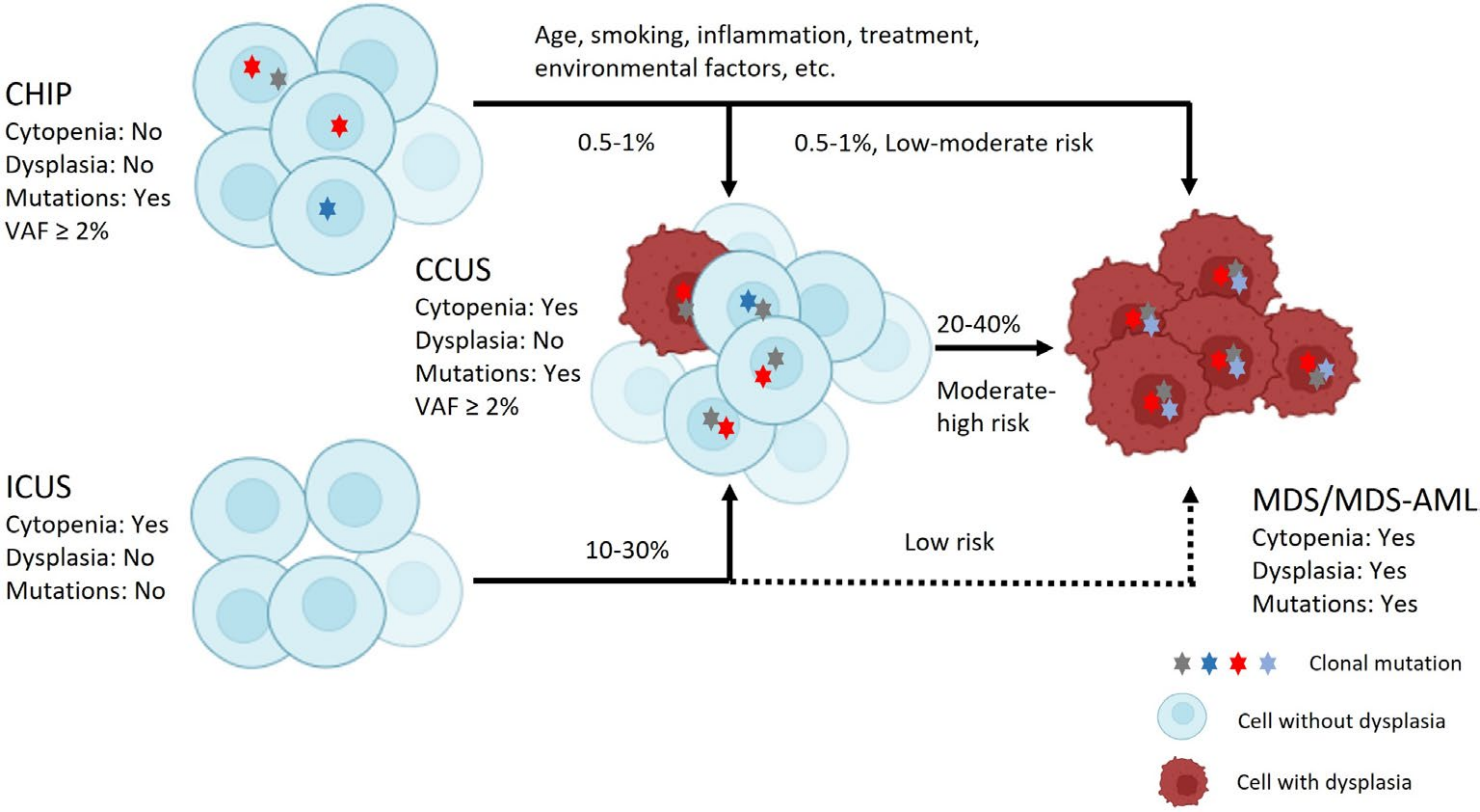
Continuum from clonal hematopoiesis to myeloid neoplasms and associated risk. This schematic depicts the relationship among states of CH and their risk of progression. From left to right, clones arise and expand under extrinsic pressures (age, smoking, inflammation, prior treatment, environmental factors). CHIP shows somatic mutations without cytopenia or dysplasia and carries an approximately 0.5%–1% per‐year risk (low–moderate) of progression. CCUS is defined by cytopenia with no or < 10% dysplasia plus mutations with VAF ≥ 2% and shows moderate–high risk (~20%–40%), especially when clone size is larger (~10%–30% VAF) or when multiple/high‐risk mutations are present; lower‐risk paths are indicated by dashed lines. Progression culminates in myeloid neoplasms (e.g., MDS/AML) characterized by cytopenia, dysplasia, and clonal mutations. AML, acute myeloid leukemia; CCUS, clonal cytopenia of undetermined significance; CH, clonal hematopoiesis; CHIP, clonal hematopoiesis of indeterminate potential; ICUS, idiopathic cytopenia of undetermined significance; MDS, myelodysplastic syndrome; VAF, variant allele frequency.

CH refers to the disproportionate clonal expansion of one or more hematopoietic stem or progenitor cell populations in the bone marrow and blood [8, 11]. CH can be caused by somatic point mutations, small insertions or deletions, or larger chromosomal gains or losses that confer a selective growth advantage. With aging, its prevalence increases significantly, and CH is considered to be a premalignant condition in a subset of individuals [12]. Within CH, clonal hematopoiesis of indeterminate potential (CHIP) and clonal cytopenia of undetermined significance (CCUS) are classified similarly in both the fifth edition of the World Health Organization Classification of Haematolymphoid Tumours (WHO‐HAEM5) and the International Consensus Classification (ICC) as premalignant clonal conditions [13, 14]. In WHO‐HAEM5, CHIP is defined as clonal hematopoiesis harboring somatic mutations in myeloid malignancy–associated genes detected in the blood or bone marrow at a variant allele fraction (VAF) of ≥ 2% (≥ 4% for X‐linked gene mutations in males), in individuals with normal peripheral blood counts who do not meet diagnostic criteria for MDS or another hematologic malignancy. CCUS is defined as clonal hematopoiesis detected in the presence of one or more otherwise unexplained persistent cytopenias that do not meet diagnostic criteria for a defined myeloid neoplasm [15, 16, 17]. WHO‐HAEM5 further clarifies that CCUS may include cases with somatic mutations meeting CHIP criteria and/or clonal chromosomal abnormalities in patients with cytopenia but without morphologic dysplasia [18]. The ICC uses a similar framework but explicitly defines cytopenia in CCUS as persistent for at least 4 months and includes either a myeloid neoplasm driver mutation at VAF ≥ 2% or a non–MDS‐defining clonal cytogenetic aberration as evidence of clonality [19]. In both systems, cytopenia thresholds are harmonized as hemoglobin < 13 g/dL in men or < 12 g/dL in women, absolute neutrophil count < 1.8 × 10^9^/L, or platelet count < 150 × 10^9^/L. These distinctions are clinically relevant in the CAR T‐cell therapy setting, where prior cytotoxic therapy, marrow involvement by the underlying malignancy, lymphodepleting chemotherapy, and CAR T‐related inflammatory toxicity can complicate the interpretation of cytopenias and CH. In contrast, idiopathic cytopenia of undetermined significance is defined by persistent unexplained cytopenia without detectable clonality or diagnostic dysplasia (Figure 1) [11].

When using the 2% threshold, population‐based sequencing studies show that CH and CCUS are uncommon before age 50 but are present in approximately 10%–20% of individuals aged 70 years or older [6, 8, 20, 21, 22, 23, 24]. The mutational spectrum is dominated by epigenetic regulators *DNMT3A*, *TET2*, and *ASXL1*, with smaller contributions from genes involved in signaling (e.g., *JAK2*), DNA damage response (DDR) (*TP53*, *PPM1D*), and RNA splicing (*SF3B1*, *SRSF2*, *U2AF1*) [8, 15, 20, 24, 25]. While *TP53* and *PPM1D* mutations are rare in untreated older individuals, they are enriched after cytotoxic chemotherapy or radiotherapy, a pattern termed therapy‐related CH [26].

The intersection of CH and CAR T cell therapy is clinically important. CAR T candidates are typically older and heavily pretreated, and multiple series report CH in 20% to 40% of such patients, compared with approximately 1%–10% in age‐matched general populations [27, 28, 29, 30]. In this high‐risk setting, CH has been associated with increased severity of inflammatory toxicities, delayed hematopoietic recovery, and a higher incidence of t‐MNs, although effects on initial response rates are generally limited or absent across cohorts. Risk may differ by mutation class: *DNMT3A* or *TET2* variants have been linked to heightened inflammatory cytokine production in experimental systems [31, 32], whereas *TP53* or *PPM1D* variants are associated with impaired marrow reserve and greater propensity for malignant evolution [26, 27]. Common CH genes and clinical correlates in CAR T cell therapy are summarized in Table 1.

**TABLE 1:** CH drivers and clinical correlates in CAR T cell therapy.

Gene	Function	Frequency in CH	Frequency pre‐CAR T	Frequency change post‐CAR T	Effect on CAR T cell therapy			References
					Cytopenia	CRS/ICANS	t‐MN	
*DNMT3A*	Epigenetic regulation	40%–60%	~25%–30%	Stable or slightly increased	Low risk	Higher risk; controversial results	Low‐moderate risk	[8, 29, 35, 76, 77, 78]
*TET2*	Epigenetic regulation	15%–25%	~10%	Stable or slightly increased	High risk	Higher risk	Low‐moderate risk	[76, 79, 80, 81, 82]
*ASXL1*	Epigenetic regulation	10%–20%	< 10%	Stable or slightly increased	Low risk	Higher risk, controversial results	Moderate risk	[35, 76, 79]
*TP53*	DNA damage repair	< 1%	~20%	Most likely expand	High risk	Low risk	High risk	[64, 83, 84]
*PPM1D*	DNA damage repair	0.5%–1%	~25%–30%	Most likely expand	High risk	Low risk	High risk	[76, 85, 86]
*SRSF2*/*SF3B1*/*U2AF1*	Spliceosome machinery	1%–2%	No data	No data	No evidence	No evidence	High risk	[84]

Abbreviations: CAR T, chimeric antigen receptor T cells; CH, clonal hematopoiesis; CRS, cytokine release syndrome; ICANS, immune effector cell‐associated neurotoxicity syndrome; t‑MN, therapy‑related myeloid neoplasms.

In this review, we synthesize the current evidence on CH in the context of CAR T cell therapy. We describe CH epidemiology in the general population and among CAR T recipients; discuss mechanisms underlying its frequency and clonal dynamics around lymphodepletion and post‐infusion inflammation; examine effects on efficacy, toxicities, and secondary malignancies; and outline clinical implications, including the potential role of pre‐therapy CH screening for risk stratification and patient management.

## 2 Epidemiology of CH

The prevalence of CH varies across healthy populations, disease cohorts, and patients exposed to genotoxic therapy. Large population sequencing studies has established a clear age gradient. In a multi‐cohort whole‐exome analysis of 17 182 participants from the United States and Europe, CH was rare before midlife and increased to 9.5% at ages 70–79, 11.7% at 80–89, and 18.4% at 90–108, with *DNMT3A*, *TET2*, and *ASXL1* predominating [8]. A complementary whole‐exome study of 12 380 Swedish individuals reported approximately 10% prevalence over age 65 versus about 1% under age 50, again dominated by *DNMT3A*, *TET2*, and *ASXL1* [24]. Deep targeted sequencing of 2530 hematologically normal French‐Canadian adults aged 55–101 found driver‐positive CH in 13.7%, 93% of which involved *DNMT3A* or *TET2* [20]. Whole‐genome sequencing (WGS) in Iceland (11 262 individuals) showed CH in 12.5% overall, with prevalence exceeding 50% after age 85 when CH was defined by WGS [21].

Beyond healthy cohorts, disease‐specific series highlight enrichment and exposure‐shaped spectra that are relevant in the context of cardio‐oncology and for patients on a trajectory toward cellular therapies. In young (≤ 60 years) ischemic stroke patients across Germany and the USA (*n* = 248), CH was detected in 21% overall; using uniform VAF thresholds and external comparators, prevalence was 7.7% versus 2.6% (Nijmegen controls) and 11.9% versus 4.1% (UK Biobank) after sensitivity adjustment, with *DNMT3A* (approximately 69%) and *TET2* (approximately 12%) predominating [33]. In oncology clinics, previous radiation, platinum, and topoisomerase‐II exposures are strongly associated with CH in DDR genes (*PPM1D*, *TP53*, *CHEK2*), and such clones preferentially expand under continued therapy [26, 34]. This “therapy‐related CH” contrasts with age‐related CH by favoring DDR lesions over epigenetic regulators, a distinction that becomes crucial in CAR T candidates.

Pre‐CAR T cell cohorts show both higher prevalence of CH and a therapy‐skewed mutational spectrum. In a series of 154 patients with lymphoma or myeloma treated with CAR T cell therapy, CH (VAF ≥ 2%) was present in 48%; in patients younger than 60 years, CH was associated with higher complete response rates and higher CRS severity, but was not associated with differences in progression‐free or overall survival (OS) [30]. In a single‐center cohort of relapsed/refractory aggressive B‐cell lymphoma, targeted deep sequencing detected CH in 34% (11/32) of patients prior to CAR T cell therapy; CH carriers had similar CRS/ICANS rates and response, and numerically better OS in that small cohort [27]. In a larger multicenter study across Germany and Austria using error‐corrected sequencing, clonal hematopoiesis at infusion was identified in 56.4% of patients at a VAF of at least 1%, with *PPM1D* the most frequently affected gene followed by *DNMT3A*, *TET2*, *ASXL1*, and *TP53*, and acute toxicities did not differ by CH status [35].

Importantly, prevalence estimates are not directly interchangeable. Rates depend on the sequencing assay type (whole‐exome versus deep targeted versus whole‐genome), panel content (inclusion of DDR or spliceosome genes), error correction and unique molecular identifier usage, and VAF thresholds (e.g., 1% vs. 2%). Studies also differ in design, sampling time point relative to cytotoxic exposures, and previous therapy profiles such as radiation or type of chemotherapy. These parameters should be considered when comparing reports or when translating prevalence data into screening policies for CAR T programs [34, 35].

In sum, CH is uncommon before age 50 but frequent thereafter, and previous therapy enriches for DDR mutations such as *PPM1D* and *TP53*. CAR T candidates present with both a higher baseline prevalence and a distinct mutational spectrum. These epidemiologic features prompt the mechanistic and clinical questions addressed in Section 3.

## 3 Dynamics of CH After CAR T Cell Therapy

**FIGURE 2:**
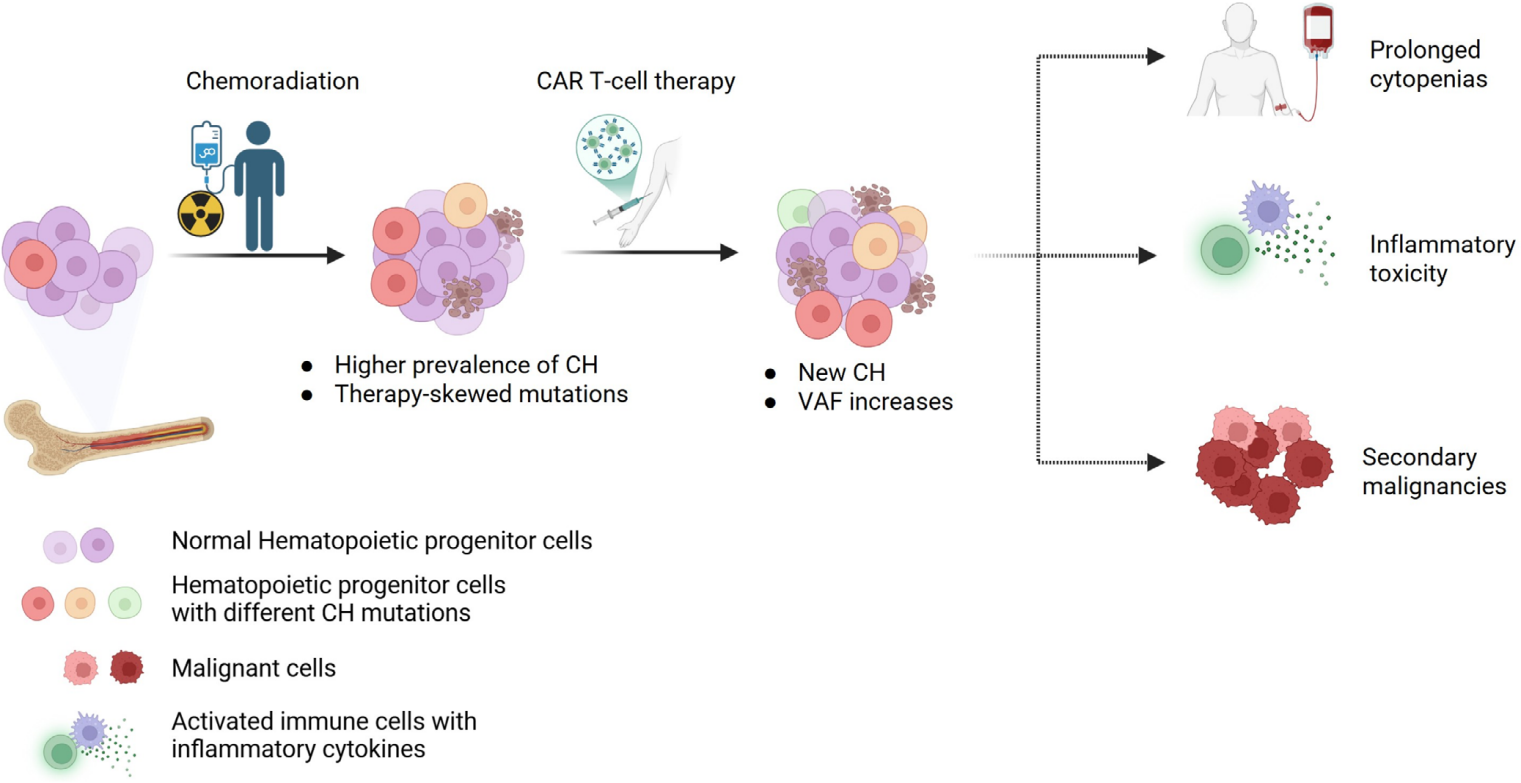
Anti‐cancer therapies and immune stress select and expand CH clones, shaping clinical outcomes. Hematopoietic stem and progenitor cells are exposed to chemoradiation and CAR T‐cell therapy, which associate with a higher prevalence of CH and therapy‐skewed mutations. After therapy, new CH clones appear and/or VAF increases in pre‐existing clones. These therapy‐shaped clones link to three downstream consequences: Prolonged cytopenias, inflammatory toxicity, and secondary malignancies (e.g., therapy‐related myeloid neoplasms). Solid arrows indicate temporal flow; dotted arrows indicate potential downstream effects. CAR, chimeric antigen receptor; CH, clonal hematopoiesis; VAF, variant allele frequency.

Population and disease cohorts show that CH prevalence rises with age and is further enriched in patients exposed to cytotoxic therapy. In CAR T cohorts, this enrichment is striking: beyond the familiar age‐related drivers (*DNMT3A*, *TET2*, *ASXL1*), there is a disproportionate representation of DDR genes such as *PPM1D* and *TP53* [26, 34]. Section 2 summarized prevalence across general and cancer populations and at infusion. Here, we focus on why CH is frequent and dynamic after CAR T: selection from prior therapies and lymphodepleting conditioning, post‐infusion inflammatory stress, and, in rare instances, direct effects of CH‐related gene perturbations within CAR T cells (Figure 2).

### 3.1 Previous Therapy and Conditioning Impose Strong Selection

Large clinical sequencing studies link previous chemotherapy and radiation to therapy‐related CH enriched for DDR genes, while untreated individuals more often carry *DNMT3A*/*TET2* [26, 34]. In therapy‐related acute myeloid leukemia/myelodysplastic syndrome (AML/MDS), truncating *PPM1D* mutations are common and confer a chemoresistance phenotype in hematopoietic stem and progenitor cells (HSPCs) under cytotoxic stress [36]. Autologous stem cell transplantation (ASCT), frequently used before CAR T in myeloma but now less common in lymphomas, similarly reveals that CH at transplant associates with inferior survival and excess t‐MN, reinforcing the concept that intensive cytotoxic exposure selects for therapy‐resistant clones [37, 38].

### 3.2 Lymphodepletion and Post‐Infusion Inflammation Drive Further Expansion

Cyclophosphamide/fludarabine lymphodepletion induces profound hematopoietic stress. In clinical and murine adoptive T cell models, peripheral blood mononuclear cells reach a nadir after cyclophosphamide/fludarabine, with a disproportionate rise in CD11b^+^ myeloid cells that suppress T cell proliferation; mobilized HSPCs display a myeloid‐biased differentiation pattern [39]. After infusion, severe CRS, and sometimes ICANS, is associated with elevated IL‐1β, IL‐6, and other cytokines that suppress hematopoiesis and can prolong cytopenias [7, 40]. CH carriers often have higher baseline IL‐1β/IL‐6, and *TET2*‐deficient macrophages oversecrete these cytokines upon inflammasome activation [31, 41]. Longitudinally, CH clones in anti‐CD19 CAR T recipients can expand within ~100 days and new mutations can emerge; about 45% of post‐infusion events fall in DDR genes, with low‐VAF *PPM1D* the most frequent [35]. In an independent CD30 CAR T cohort, initially small clones (VAF < 1%) expanded by a mean 3.37‐fold and could be tracked for years; matched CH clones were detectable at low magnitude in the infused cell product but did not alter product phenotype or transduction efficiency, suggesting that systemic stress and inflammation, rather than the product itself, drove expansion [42].

Together, cumulative exposure plus lymphodepleting conditioning shifts the clonal landscape toward DDR‐mutant clones that better tolerate cytotoxic and replicative stress [26, 43].

### 3.3 CH Mutations Can Modulate CAR T Cell Behavior

A landmark case described biallelic *TET2* disruption in a patient's CAR19 cells, with dominant central‐memory CAR T expansion and durable tumor control, accompanied by high‐grade CRS [44]. Vector insertion in proximity to *CBL*, which encodes an E3 ubiquitin‐protein ligase that negatively regulates T cell receptor signaling, has also been linked to clonal CAR T expansion, resulting in complete remission without worsened CRS [45]. Preclinical editing of *DNMT3A* reduces exhaustion and improves antitumor activity in CAR T cells [46]. These observations illustrate a plausible, cell‐intrinsic axis through which CH‐related pathways can influence CAR T biology.

### 3.4 Synthesis and Implications

Taken together, age, cumulative genotoxic exposure, lymphodepleting chemotherapy, and post‐infusion inflammation create a strong selection environment that disadvantages wild‐type hematopoietic stem cells and favors DDR‐mutant, inflammation‐prone clones. In parallel, CH‐gene perturbations within CAR T cells can reinforce expansion and persistence. These interlocking mechanisms explain the high frequency and dynamic behavior of CH in the CAR T setting and support prospective evaluation of CH in trial design, toxicity‐mitigation research, and structured long‐term surveillance.

## 4 Clinical Significance of CH in CAR T Cell Therapy

Building on Section 3, two clinical questions arise: does pre‐existing CH affect antitumor efficacy, and does it modify toxicities and long‐term outcomes after CAR T? Current data across anti‐CD19 and anti‐B‐cell maturation antigen (BCMA) products show no consistent decrement in response or survival, while suggesting gene‐ and context‐specific effects on inflammatory toxicities, cytopenias, and secondary myeloid neoplasms (Figure 2) [28, 47].

### 4.1 Efficacy

Whether baseline CH alters CAR T antitumor efficacy remains unsettled, but the weight of evidence shows no consistent decrease in response or survival, with gene‐ and context‐specific exceptions. Outside the CAR T setting, several lines of evidence suggest that CH correlates with inferior outcomes in cancer populations: in 8810 patients with solid tumors profiled by MSK‐IMPACT, therapy‐related CH (enrichment for DDR genes) was common and associated with worse OS, independent of overt myeloid neoplasm transformation [34]. In newly diagnosed high‐grade B‐cell lymphoma, CH at presentation predicted inferior progression‐free and OS [48]. After ASCT for lymphoma, CH tracked with worse OS and higher t‐MN risk [37], whereas a national cohort showed that DDR‐gene CH (*PPM1D*, *TP53*, *RAD21*, and *BRCC3*)—rather than CH overall—drove inferior OS [49]. In DLBCL treated with standard therapies, CH was frequent; overall CH was not independently prognostic after age adjustment, but larger clones (VAF ≥ 5%) or multiple CH mutations trended to worse OS [50].

In contrast, in the patients who received CAR T cell therapy, there is not a consistently observed negative effect of CH on OS. In 154 patients with lymphoma or myeloma receiving CD19‐ or BCMA‐directed products, CH (48%) did not worsen progression‐free survival and OS, and in patients < 60 years CH was actually associated with higher CR rates [30]. In a multicenter series using error‐corrected sequencing at CAR T infusion, investigators found frequent DDR mutations but no clear decrement in early efficacy outcomes attributable to CH [35]. Data in myeloma with BCMA CAR T similarly suggest little to no effect of CH on progression‐free survival and OS, though sample sizes remain modest [51].

### 4.2 Inflammatory Toxicities

CRS and ICANS are driven by broad cytokine cascades in which IL‐6 and IL‐1 are central but not exclusive mediators; other cytokines implicated include IFN‐γ, GM‐CSF, and TNF‐α, among others. Grading should follow the American Society for Transplantation and Cellular Therapy (ASTCT) consensus criteria for uniformity across trials and practice [40].

DTA‐class CH (i.e., *DNMT3A*, *TET2*, and *ASXL1*) is linked to a pro‐inflammatory myeloid phenotype. In human population studies, CH correlates with elevated inflammatory signaling and cardiovascular events [52]. Complementary murine work shows that *TET2*‐deficient macrophages have heightened NLRP3 inflammasome activation with increased IL‐1β and IL‐6 production, indicating a primed myeloid state that could amplify CAR T toxicities [31, 41].

Clinically, associations between CH and inflammatory toxicity are variable. In a single‐center cohort of 62 recipients of CD19 or BCMA CAR T cells, CH (24% prevalence) was associated with grade ≥ 2 CRS in 60% vs. 28% without CH (OR 3.9, 95% CI 1.2–13.2), with no difference in ICANS, progression‐free survival, and OS [53]. In a multicenter series of 110 anti‐CD19 CAR T recipients at infusion, CH was present in 56.4% and enriched for *PPM1D* and *TP53*; CRS and ICANS rates did not differ by CH status, though VAFs often rose and new mutations (*TP53, STAT3* and *GATA2*) emerged on follow‐up [35]. In a 154‐patient series of lymphoma and myeloma, CH (48%) was associated with higher complete response and higher CRS severity only among patients younger than 60 years, with no difference in progression‐free or OS overall [30]. These heterogeneous signals align with broader observations that CH associates with dysregulated inflammatory programs and outcomes in solid tumors [54].

Overall, current data support CH as a risk modifier rather than a deterministic driver of inflammatory toxicity. Variability across studies likely reflects differences in assay methodology (VAF thresholds, error correction), sampling relative to lymphodepletion, CAR T product design, institutional practices for bridging and early cytokine blockade, and the distribution of clone classes (particularly DTA vs. DDR lesions) and sizes.

### 4.3 Cytopenias and Marrow Failure

Prolonged cytopenias after CAR T are common and clinically relevant, affecting infection risk, transfusion needs, and access to subsequent therapy. Baseline inflammation and marrow reserve at the time of lymphodepletion predict both early (day 0–30) grade ≥ 3 cytopenias and late cytopenias beyond day 60, as captured by validated hematotoxicity scores such as CAR‐HEMATOTOX [55].

Evidence linking therapy‐selected CH, particularly in DDR genes, to delayed hematopoietic recovery after CAR T is increasing, although results vary across cohorts. At infusion, CH is frequent and often enriched for *PPM1D* and *TP53*. On serial sampling within approximately 100 days, about 45% of post‐infusion clonal expansions involve DDR genes, with *PPM1D* common at low VAFs [28]. In that multicenter series, however, prolonged cytopenias were not statistically associated with baseline CH [35]. By contrast, in BCMA CAR T for myeloma, CH has been associated with a higher risk of prolonged cytopenias without decrements in progression‐free survival or OS [51].

Mechanistic plausibility for impaired recovery with DDR‐mutant CH is strong in the ASCT setting, which imposes comparable cytotoxic stress. In lymphoma patients undergoing ASCT, CH at transplant (approximately 14%) was linked to inferior OS (hazard ratio about 1.6) and progression‐free survival (hazard ratio about 1.45), largely through excess tMNs; *TP53* and *PPM1D* mutations were over‐represented among these t‐MN cases [37]. In lymphoma ASCT, the presence of CH in the stem‐cell product correlated with delayed platelet and neutrophil recovery and worse survival, with *PPM1D* as a recurrent driver [56]. In myeloma ASCT, grafts harboring *DNMT3A* or *PPM1D* mutations showed reduced stem‐cell yields and delayed platelet recovery in high‐resolution longitudinal modeling [57]. At the cellular level, truncating *PPM1D* mutations confer a chemoresistance phenotype in HSPCs with abrogated DDR signaling, reduced apoptosis, and selective expansion under cytotoxic stress; these effects are reversible with an allosteric *PPM1D* inhibitor [43].

Overall, lymphodepleting chemotherapy and post‐infusion inflammation create a selective niche that disadvantages wild‐type hematopoietic stem cells and favors DDR‐mutant clones. When CH is dominated by *PPM1D* and *TP53*, the risks of prolonged thrombocytopenia and neutropenia appear greater, particularly with BCMA CAR T. Cohorts profiled at CD19 infusion show weaker or null associations, likely reflecting differences in product, disease biology, assay sensitivity, VAF thresholds, and sampling relative to hematotoxic insults. These hematopoietic risks also may foreshadow secondary myeloid neoplasms, which are addressed in Section 4.4.

### 4.4 Secondary Malignancies

Delayed hematopoietic recovery carries clinical significance beyond cytopenia itself, as it can herald the emergence of t‐MNs. Among the most concerning long‐term complications after CAR T are MDS and AML [58, 59, 60].

Incidence varies by cohort and follow‐up duration. In adults receiving commercial anti‐CD19 CAR T for B‐cell lymphoma at a single center (*n* = 159), the cumulative incidence of t‐MN was approximately 5% at 1 year and 11% at 2 years [58]. In an institutional series with longer follow‐up (median 38 months) across lymphomas and myeloma (*n* = 246), second primary malignancies (SPMs) occurred in 8.5%, with hematologic SPMs dominated by MDS/AML [59]. In a cohort of 213 myeloma patients treated with BCMA‐directed CAR T, 9% developed secondary myeloid neoplasms, most often therapy‐related MDS driven by expansion of *TP53*‐mutated CH [61]. A meta‐analysis of 5517 patients confirmed that SPMs, including myeloid neoplasms, are clinically relevant long‐term events after CAR T [60]. A two‐center study in B‐cell lymphoma (*n* = 355) estimated a 3‐year cumulative incidence of any SPM of approximately 14%, including solid tumors (6%), skin cancers (4%), and hematologic malignancies (4%) [62]. Analysis of SPMs that were reported in 536/12394 (4.3%) adverse event after CAR T cell therapy in the FDA administration adverse event reporting system revealed that myeloid neoplasms represented the most frequent event (62.1% of all SPMs and 2.7% of all CAR T reports), which include 38.8% MDS, 19.8% AML and 2 cases of T cell large granular lymphocytic leukemia [63]. Several reports describe t‐MNs arising within 1 to 2 years after CAR T, earlier than the typical 5 to 7 years after conventional alkylator chemotherapy and radiotherapy [58, 64].

t‐MNs after CAR T often arise from pre‐existing CH with gene‐specific enrichment. In a multicenter French analysis of B‐cell lymphoma treated with CD19 CAR T (*n* = 539), 85.7% of t‐MNs harbored antecedent driver mutations, most commonly *TP53* [64]. Case series and institutional cohorts similarly trace post‐CAR T t‐MN to antecedent CH, with recurrent *TP53*, *PPM1D*, and spliceosome genes recurrently affected [65]. Oncologic therapy selects for DDR clones, and truncating *PPM1D* mutations confer chemoresistance that enables outgrowth under cytotoxic stress [26, 43].

Lymphodepletion with cyclophosphamide/fludarabine, post‐infusion inflammation, infections, and repeated hematopoietic stress create a niche that impairs normal progenitors and favors DDR‐mutant clones, consistent with clonal expansion patterns during the first 100 days after infusion [26, 43]. While supportive care such as G‐CSF is often necessary, human data directly implicating G‐CSF in CH expansion are limited. It is more accurate to view growth‐factor support as part of a broader regenerative and inflammatory context that may indirectly influence clonal dynamics [57, 66, 67]. In individual cases, the same driver detected before CAR T has been documented at leukemic transformation, reinforcing a model of selection rather than de novo mutagenesis [65].

Secondary T cell lymphomas are rare after CAR T. One study reported a lethal T cell lymphoma without evidence of oncogenic vector integration and outlined methods for clonal relationship testing and viral‐vector monitoring, supporting lifelong surveillance while emphasizing the rarity of this event [68].

Incorporating CH assessment into pre‐CAR T evaluation may refine risk stratification for secondary myeloid neoplasms. Practical steps include (i) targeted sequencing for myeloid driver genes (with particular attention to *TP53/PPM1D* and spliceosome genes); (ii) intensified post‐infusion monitoring in patients with high‐risk CH or persistent grade ≥ 3 cytopenias; and (iii) early bone‐marrow reassessment to distinguish immune effector cell–associated hematotoxicity from evolving t‐MN. Programs should prospectively track clone size (VAF) and composition over time and integrate these data with clinical scores and inflammatory biomarkers. Where feasible, enrollment in registries and prospective studies should be prioritized to define absolute and gene‐specific risks and to test mitigation strategies [58, 60, 62].

## 5 Clinical Implementation

Evaluating myeloid mutations before treatment is increasingly important because of the clinical implications of CH dynamics. This is particularly true for older patients and those with extensive previous cytotoxic therapy, as they have a higher prevalence of pre‐existing CH.

Risk stratification tools, such as the CAR‐HEMATOTOX score and the immune effector cell‐associated hematotoxicity (ICAHT) grading system, can help predict patient outcomes [67]. Biological factors also play a role; perturbations in the immune system, such as T‐ or NK‐cell dysfunction, and chronic inflammatory states likely contribute to ICAHT [69, 70]. These conditions may also promote the development of MDS, including subtypes with mutations, such as *PPM1D*.

Although cases of t‐MDS after CAR T cell therapy have been reported, data on pre‐existing, predisposing myeloid mutations in the bone marrow remain limited [58]. Therefore, systematically monitoring for CH‐associated mutations could clarify their role in the evolution of t‐MN. Further clinical datasets and mechanistic studies are required to establish the value of incorporating pre‐ and post‐CAR T marrow screening into routine practice.

### 5.1 Pre‐Infusion Screening

Pre‐infusion genotyping must have a clear clinical purpose: to identify CH features that could alter peri‐infusion management or early follow‐up strategies. For most programs, testing peripheral blood prior to lymphodepleting chemotherapy may be the appropriate approach.

When substantial bridging chemotherapy or radiotherapy occurs between the initial evaluation and conditioning, repeat sampling may be considered close to conditioning per institutional practice or within research protocols. Bridging therapies can select for *DDR*‐mutant clones and change their size over short intervals [26]. The clinical yield of testing may be greater in patients with high risk, such as those with a history of ASCT or heavy exposure to platinum or topoisomerase II inhibitors, who have a higher pretest probability of *DDR*‐mutant CH [26, 71].

The gene panel content should cover several key categories: the canonical epigenetic regulators (*DNMT3A*, *TET2*, *ASXL1*), DDR genes (*TP53*, *PPM1D*, *CHEK2*), and spliceosome genes (*SF3B1*, *SRSF2*, *U2AF1*). *JAK2* is also commonly included given its therapeutic and diagnostic implications. Where available, expanded panels that include *RUNX1*, *CBL*, and *BCOR* can provide additional context without materially increasing complexity.

Interpretation should follow the standard definition of CH, namely one or more myeloid driver mutations at a variant‐allele fraction of at least 2% in individuals without cytopenia (noting the confounding effects of therapy and/or conditioning) or hematologic malignancy. However, it is important to recognize that smaller clones detected with error‐corrected sequencing can also be clinically informative. Therefore, findings should be weighed by gene class and clone size rather than by a single binary cutoff [16, 72, 73].

### 5.2 Risk Stratification

While genotype alone is insufficient to guide management, it can significantly enhance existing clinical frameworks. Scores such as CAR‐HEMATOTOX, which quantify baseline inflammation and marrow reserve to predict cytopenias, could be augmented with CH information. This combination could allow the refinement of a patient's risk profile to establish earlier triggers for intervention [55, 74].

In practice, risk can be stratified more effectively. A patient without CH, or with only a single small DTA clone and a low CAR‐HEMATOTOX score, is typically monitored on standard schedules. The risk level increases when DTA clones are larger or multiple. It rises more clearly when DDR mutations are present, even at modest variant allele fractions. This is particularly true for patients with antecedent cytotoxic exposures or a history of ASCT [26, 71]. Observational data from lymphoma and myeloma cohorts support this pattern. While the presence of CH did not consistently worsen progression‐free survival or OS, the specific subtype of DDR‐mutant CH was associated with adverse outcomes. These included delayed hematologic recovery and, when combined with post‐infusion inflammation, a higher likelihood of both clonal expansion and t‐MNs [51, 58, 73, 75]. Consequently, programs that formalize risk tiers could combine multiple factors: gene class, clone size, prior exposure history, and the CAR‐HEMATOTOX score. This integrated approach could allow for prospectively specifying laboratory follow‐up intervals and defining clear thresholds for bone marrow reassessment.

### 5.3 Peri‐Infusion Management

The peri‐infusion period offers an opportunity to preempt avoidable complications without compromising efficacy. Management of CRS and ICANS should follow institutional pathways based on ASTCT criteria.

Hematologic recovery after CAR T is variable. Although associations with DDR‐mutant CH have been described, the extent to which CH predicts delayed recovery remains uncertain. This consideration is particularly relevant for patients receiving BCMA‐directed products or those with a history of intensive antecedent therapy. Growth factor support can be initiated after clinically significant CRS has resolved, following institutional practice. It is worth noting that human data directly linking G‐CSF to CH expansion are limited; therefore, its use is best considered within the broader context of post‐infusion marrow recovery [57, 66].

For patients who develop sustained grade 3 or higher cytopenias or bi‐lineage involvement, an early bone marrow reassessment is advisable. This could help distinguish immune effector cell‐associated hematotoxicity from an evolving t‐MN and allows for timely supportive measures. Select high‐risk patients, particularly those with DDR‐mutant CH and previous ASCT, have undergone a stem cell boost in some settings, although the evidence remains largely observational and practices vary among programs [55, 58, 71].

CAR T programs are already protocolized. The key question is whether there is sufficient data to support routine CH assessment before and after CAR T. It would be reasonable, within research protocols or institutional quality‐improvement efforts, to consider baseline and serial CH assessments to characterize clonal dynamics and explore associations with outcomes. Where feasible, prospectively recording clone size and composition within such frameworks could inform future protocol development, or at least spur future research.

## 6 Conclusion

The intersection of CH and CAR T cell therapy reveals a complex yet clinically significant relationship influencing therapeutic efficacy, adverse event profiles, and long‐term hematologic health. Enhancing our understanding of CH dynamics and mutation‐specific effects on CAR T therapy may inform risk assessment and targeted therapeutic strategies. Future clinical trials and translational studies incorporating CH profiling may enhance precision‐oncology approaches in the evolving landscape of CAR T cell immunotherapy.

## Author Contributions

W.D. drafted the manuscript and prepared the figures and table. S.K. drafted and edited the manuscript. S.G., G.Y., and A.B. critically reviewed the manuscript and provided comments. All authors read and approved the final version of the manuscript.

## Funding

The authors have nothing to report.

## Ethics Statement

This article is a review and does not report any original studies involving human participants or animals performed by the authors. Ethics approval and informed consent were therefore not required.

## Conflicts of Interest

The authors declare no conflicts of interest.

## Data Availability

Data sharing not applicable to this article as no datasets were generated or analyzed during the current study.
